# Embolic Cerebrovascular Accident Secondary to Device-Related Thrombus Post WATCHMAN Device Implantation

**DOI:** 10.7759/cureus.26892

**Published:** 2022-07-15

**Authors:** Palak Patel, Nagapratap Ganta, Giuseppe Filice, Ivan Richard, Frederick Acquah, Dina Alnabwani, Harshil B Patel

**Affiliations:** 1 Internal Medicine, Hackensack Meridian Health Ocean University Medical Center, Brick, USA; 2 Cardiology, Hackensack Meridian Health Ocean University Medical Center, Brick, USA

**Keywords:** left atrial thrombus, anticoagulation, left atrial appendage closure (laac), atrial fibrillation (af), watchman device, embolic cerebro vascular accident (embolic cva), device related thrombus (drt)

## Abstract

Atrial fibrillation (AF) is one of the most common cardiac arrhythmias encountered. Aggressive and appropriate management, along with identification and modification of risk factors, remains at the forefront of evidence-based practice. Thrombus formation (primarily in the left atrial appendage) and consequent thromboembolism are risks associated with AF. Anticoagulation is utilized to prevent and reduce AF-induced complications such as stroke, heart failure, and death. However, in instances when the risk of bleeding from anticoagulation outweighs the benefits of stroke prevention, other modalities such as left atrial appendage closure (LAAC) devices like the WATCHMAN device (Boston Scientific, MA) are utilized. LAAC devices, such as the WATCHMAN device, are also not without significant risks, one of them being device-related thrombus (DRT) formation. We present a case of device-related thrombus formation post WATCHMAN implantation and a subsequent embolic cerebrovascular accident (CVA).

## Introduction

Atrial fibrillation (AF) is an irregular heart rhythm and the most common cardiac arrhythmia, affecting almost 0.5% of the world’s population and 0.4% to 1% of the United States population. By 2050, it has been estimated that 6-12 million people will be diagnosed with this condition in the US [1]. AF increases a patient’s risk of life-threatening events and conditions, including stroke, heart failure, and death. The risk of stroke on average is five times higher [2] than the average population and is a serious complication of AF that is not only associated with long-term disability and mortality but also a significant economic burden. The American College of Cardiology (ACC) and the American Heart Association (AHA) recommend the use of a scoring system called the CHA2DS2-VASc (congestive heart failure, hypertension, age ≥75 (doubled), diabetes, stroke (doubled), vascular disease, age 65 to 74 and sex category (female)) score in patients with nonvalvular atrial fibrillation to determine the need for anticoagulation to prevent stroke [3]. Anticoagulation is recommended for patients with one or more non-sex CHA2DS2-VASc stroke risk factors (score of >1 in men or >2 in women). However, anticoagulation comes with its own risk, as it predisposes patients to bleeding, especially intracranial hemorrhage and gastrointestinal (GI) bleeding [4]. Since the left atrial appendage (LAA) is the main source of thrombi formation in 90% of patients with nonvalvular atrial fibrillation, another approach to stroke prevention is to physically block clots from exiting the LAA [5]. In patients with non-treatable, recurrent, or major bleeding, a left atrial appendage (LAA) occluding device or surgical LAA occlusion may be considered. However, a thrombus may develop on the left atrial side of the device and can subsequently embolize [6]. The WATCHMAN left atrial appendage closure device from Boston Scientific (MA) was approved by the United States Food and Drug Administration (USFDA) for the purpose of reducing the risk of thromboembolic events. Although as effective as warfarin in lowering rates of ischemic stroke [7], the device is not without complications of its own. Data suggest possibilities of pericardial effusion/tamponade, periprocedural stroke secondary to air embolism, a peridevice leak, device migration, and device-related thrombosis (DRT) [8]. We present a case of a patient with ischemic stroke secondary to device-related thrombus after WATCHMAN device implantation for AF after recurrent bleeding from anticoagulation.

## Case presentation

A 75-year-old female with a past medical history of atrial fibrillation status post (s/p) WATCHMAN device placement presented to the emergency department with complaints of left-sided weakness, left-hand numbness/tingling, nausea, and vomiting. Other significant history included chronic kidney disease stage 3a, congestive heart failure with preserved ejection fraction (HFpEF), hypertension, hyperlipidemia, obesity, history of hematuria, history of breast cancer s/p chemotherapy and bilateral left mastectomy, and arthritis. Upon presentation, the patient reported recently starting on Yttrium-90 radiotherapy treatment two months prior to presentation for the treatment of metastatic cholangiocarcinoma to the liver. The patient reported her second Y-90 treatment was two days prior to presentation and that she usually experiences nausea post-treatment. One day prior to presentation, the patient reported transient left arm weakness, numbness, and tingling lasting approximately 30 minutes in duration, which prompted her to seek medical care at a different facility. She underwent computed tomography (CT) of the head and cervical spine, which were unremarkable, and she was discharged home. On the day of admission, she again experienced the same symptoms and, in addition, was hypotensive, prompting her to come to the emergency room. Of note, the patient also had a fall six weeks prior to presentation and sustained a left trimalleolar fracture, requiring open reduction internal fixation and splint placement.

ROS was positive for numbness of the left hand, left ankle pain, and nausea. Vitals signs were temperature 98.8 degrees Fahrenheit, blood pressure 106/70, pulse 100 beats per minute, respiratory rate 18, and pulse oximetry 98% on room air. On physical examination, the patient appeared to be in mild distress. She was found to have an irregularly irregular rhythm and a systolic murmur, had scattered rhonchi to auscultation bilaterally, and a splint on the left lower extremity with a good capillary refill. Pertinent laboratory findings were hemoglobin 12.1 g/dL (13.2-17.5 g/dL), white blood cell count 11.4x10^3/uL (4.5-11.0x10^3/uL), platelet count 244x10^3/uL (140-450x10^3/uL, and internalized normalized ratio of 1.07 (0.88-1.15). Comprehensive metabolic panel showed a blood urea nitrogen of 27 mg/dL (5-25 mg/dL), serum creatinine of 1.34 mg/dL (0.61-1.24 mg/dL), sodium of 133 mmol/L (136-145 mmol/L), potassium of 3.7 mmol/L (3.5-5.2 mmol/L), chloride of 97 mmol/L (96-110 mmol/L), bicarbonate of 23 mmol/L (24-31 mmol/L), glucose of 112 mg/dL (70-99 mg/dL), alanine aminotransferase of 29 U/L (10-24 U/L), aspartate aminotransferase of 55 U/L (10-60 U/L), alkaline phosphatase of 240 U/L (38-126 U/L), and a total bilirubin of 1.0 mg/dL (0.2-1.3 mg/dL). Other significant lab work included brain natriuretic peptide of 1173 pg/mL (<100 pg/mL) and troponin of 0.24 ng/mL (<0.04 ng/mL).

**Figure 1:**
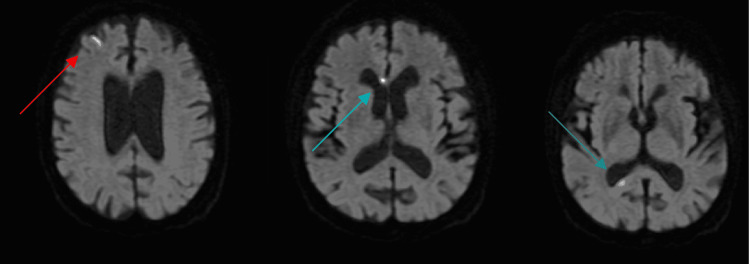
MRI of the brain revealing scattered, small, right-sided frontal (red arrow) and paraterminal gyrus (blue arrow), cortically based acute to subacute infarcts without associated hemorrhage or edema, and a subacute infarct to the right corpus callosum (green arrow)

**Figure 2:**
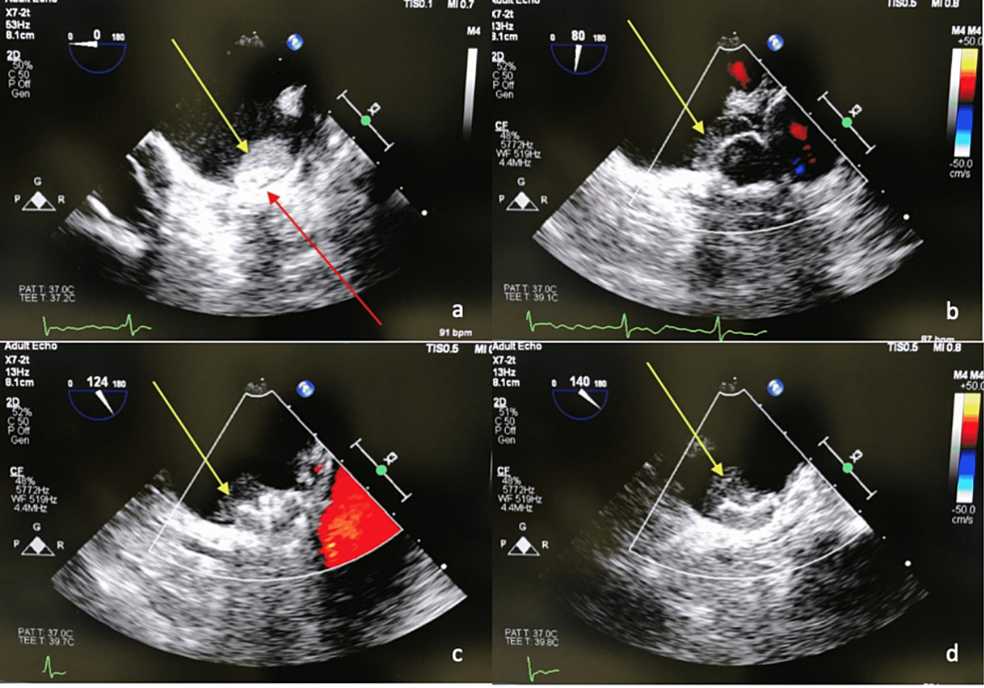
Transesophageal echocardiogram illustrating the presence of a thrombus (yellow arrow) a. Appendage view at 0° with a visible view of the WATCHMAN device crown (red arrow). b. Appendage view at 80° c. Appendage view at 124°. d. Appendage view at 140°.

Urinalysis was unremarkable except for small blood. EKG showed A-fib with a rapid ventricular response (RVR) and non-specific ST and T wave abnormalities. Chest X-ray revealed right heart prominence without any acute cardiopulmonary pathology. Magnetic resonance imaging (MRI) of the brain with and without contrast (as evident in Figure 1) showed scattered, small, right-sided frontal and parietal cortically based acute to subacute infarcts without associated hemorrhage or edema and a subacute infarct to the right corpus callosum. The patient was started on aspirin 81 mg, clopidogrel 75 mg, and rosuvastatin 20 mg. A transthoracic echocardiogram (TTE) was done, which showed mildly reduced ejection fraction (EF), moderately dilated left atrium (LA), and mild mitral regurgitation (MR). The patient subsequently underwent a TEE that showed mildly dilated LA with a moderate-sized LA thrombus (as illustrated in Figure 2) measuring 1.0 X1.1 cm attached to a WATCHMAN device. The patient was treated with therapeutic-dose enoxaparin and aspirin 81 mg and eventually transitioned to apixaban 5 mg twice daily for the treatment of intracardiac thrombi due to prior intolerance of both rivaroxaban and warfarin. While inpatient, the patient underwent rigorous physical and occupational rehabilitation. After improvement of symptoms, she was eventually discharged to a subacute rehabilitation facility for strengthening, range of motion, gait, and endurance training.

## Discussion

The widespread worldwide prevalence of atrial fibrillation places a large financial burden on the healthcare field, thus making it imperative to appropriately treat and manage in a safe and cost-effective manner. A CHA2DS2-VASc score is the most common method of assessing stroke risk in patients with non-valvular AF, with those at increased risk of adverse events, including mortality, if not anticoagulated [9]. Aside from anticoagulation, an alternative therapeutic option for those at high risk for an ischemic stroke and/or contraindications to systemic oral anticoagulation is the closure of the left atrial appendage with a WATCHMAN device [10]. While evidence for long-term postprocedural anticoagulation is lacking, the short-term use of anticoagulation for thromboembolic events prevention shows some promising outcomes [11]. The PREVAIL trial showed that the WATCHMAN device was non-inferior to warfarin for the occurrence of late ischemic events such as ischemic stroke or systemic embolism [7].

Despite the obvious benefits of the LAA occluding device, it is not without significant risks like the formation of a device-related thrombus (DRT). Recently, an observational study suggested a 7% per year risk of device-related thrombosis with LAA occluder placement [12]. DRT is associated with a higher rate of stroke and systemic embolism post WATCHMAN device placement when present [6]. DRT is not associated with an increased risk of cardiovascular or all-cause mortality [13]. According to Dukkipati et al., the overall incidence of DRT is 3.7% on the following Food and Drug Administration (FDA)-approved regimen post WATCHMAN device placement: warfarin and aspirin 81 mg for six weeks, followed by clopidogrel 75 mg and aspirin 81-325 mg daily from day 45 to six months, and ultimately aspirin 325 mg from six months onward. Although nearly 75% of patients that develop DRT do not experience a stroke, anticoagulation should promptly be resumed when DRT is detected to potentially decrease the risk of ischemic stroke [6]. Most DRTs are found after 45 days; the current standard of a transesophageal echocardiogram at 45 days is not sufficient, and additional transesophageal echocardiography surveillance should be obtained following the cessation of oral anticoagulation at four or six months postimplantation [6]. Another alternative to multiple TEEs can be to delay performing the initial surveillance to longer than 45 days post-implantation to maximize the yield of discovering a thrombus. However, that would come with a caveat of its own, as it would imply prolonging the duration of anticoagulation and thus increasing the risk of bleeding, hence defeating the very purpose of the WATCHMAN device.

Our patient underwent a WATCHMAN device implantation due to an increased risk of thromboembolism (CHA2DS-VASc score of 4) and hematuria while being on rivaroxaban. The procedure was successful, and the patient was then placed on rivaroxaban 15 mg and aspirin 81 mg. A surveillance TEE was performed 45 days post-implant and showed a well-seated 20 mm WATCHMAN device in the left atrial appendage with 9-11% compressions. The patient was then taken off rivaroxaban and was started on dual antiplatelet therapy with aspirin 325 mg and clopidogrel 75 mg. However, the clopidogrel was stopped prematurely and the aspirin dose was lowered to 81 mg daily due to the increased risk of bleeding postop when the patient sustained a left ankle fracture post fall and required surgical intervention. The early cessation of clopidogrel might have been a culprit for the thrombus formation and subsequent embolic cerebrovascular accident. Some authors have evaluated whether the use of novel oral anticoagulants (NOACs) would be more effective after LAAC. When NOACs were compared to warfarin, they were found to be safe and effective for short-term anticoagulation following LAAC with the WATCHMAN device [11]. Some have suggested continuing oral anticoagulation due to its proposed superiority during the endothelization process (between 45 days and six months) after LAA occlusion to prevent thrombus formation [14].

However, in addition to the early cessation of dual antiplatelet therapy, there are other factors contributing to a prothrombotic state such as permanent atrial fibrillation, elevated CHA2DS2-VASc score, history of transient ischemic attack or stroke, vascular disease, coronary artery disease (CAD), prior venous-thromboembolic disorder, and hypercoagulability disorders [13]. Lower left ventricular ejection fraction, lower LAA emptying flow velocity, and larger LAA diameter are other structural/functional factors that predispose patients to thrombus formation due to the pooling of blood. The above-mentioned factors are likely indicative of a fibrotic, immobile atrium with a low flow state [14]. Procedural factors, such as compression and consequent deformation of the surface of the device, exposed metal at the attachment screw, and deep implantation of LAA occlusion devices with exposed/uncovered trabeculae further contribute to the development of DRT due to greater exposed surface area [14].

The DRT risk score can be calculated and categorized as none, low risk (1 point), or high risk (>2 points) based on the presence of risk factors. The low-risk category has a 1-fold increased risk of DRT formation while the high-risk category has a 2.1-fold increased risk of DRT formation when compared to those without risk factors. The scoring system is as follows for the DRT risk score - 1 point each for renal insufficiency, implantation depth >10 mm from the pulmonary vein limbus, and nonparoxysmal A.fib and 4 points each for iatrogenic pericardial effusion and hypercoagulable state [13]. Given the increased risk of thromboembolism, aggressive measures must be taken to promptly place patients on therapeutic anticoagulation with persistent monitoring and imaging to ensure resolution; however, no general consensus exists for the management of DRT post WATCHMAN implantation. Studies have shown that in patients with LAA thrombus, four weeks of warfarin therapy resulted in thrombus resolution in 63-89% when reassessed by TEE; data on thrombus resolution with NOACs have been variable with a 41.5% success rate with six weeks of rivaroxaban and 52% with five weeks of apixaban [15]. Despite these statistics, our patient was started on apixaban for the treatment of DRT due to prior intolerance of both rivaroxaban and warfarin.

## Conclusions

Atrial fibrillation has become one of the major epidemics and public health issues in the last 20 years, and it will continue to grow in the coming years. Left atrial appendage (LAA) closure with devices like the WATCHMAN appears to be a safe, effective, and promising alternative to oral anticoagulation (OAC) in the appropriate population; however, it is not without risks of its own - primarily the development of a device-related thrombus (DRT). There are several factors associated with the development of DRT and many solutions have been proposed to combat it. Failure to adhere to the currently established FDA-approved guidelines remains the strongest preventable factor. Although our patient was at increased risk for a thromboembolic phenomenon at baseline due to her multiple comorbidities, including permanent AF, high CHADS-VASc score, and active malignancy, her particular case presentation sheds light on the necessity of continuing dual antiplatelet therapy for at least six months as recommended by guidelines. Future studies should focus on the comparative safety and efficacy of different LAAC devices and on various post-procedural antithrombotic regimens, including head-to-head comparisons with NOAC, to better understand device-related thrombus in order to advocate the use of the LAA for non-valvular AF. These studies could help modify the paradigm so that LAAC is no longer considered a "last resort" treatment for AF patients who are ineligible for OAC, but rather a second or even first-line therapeutic option for select AF patients.
